# Escaping the enemy’s bullets: an update on how malaria parasites evade host immune response

**DOI:** 10.1007/s00436-023-07868-6

**Published:** 2023-05-23

**Authors:** Chinonso Anthony Ezema, Innocent Uzochukwu Okagu, Timothy Prince Chidike Ezeorba

**Affiliations:** 1 Department of Microbiology, Faculty of Biological Sciences, University of Nigeria, Enugu State, 410001 Nigeria; 2 Division of Soft Matter, Hokkaido University, Sapporo, 060-0810 Japan; 3 Department of Biochemistry, Faculty of Biological Sciences, University of Nigeria, Enugu State, 410001 Nigeria; 4 Department of Genetics and Biotechnology, Faculty of Biological Sciences, University of Nigeria, Enugu State, 410001 Nigeria; 5 Department of Molecular Biotechnology, School of Biosciences, University of Birmingham Edgbaston, Birmingham, B15 2TT UK

**Keywords:** Malaria, Host immune response, Evasion of host immunity, Rosetting, *Plasmodium* infection

## Abstract

Malaria continues to cause untold hardship to inhabitants of malaria-endemic regions, causing significant morbidity and mortality that severely impact global health and the economy. Considering the complex life cycle of malaria parasites (MPs) and malaria biology, continued research efforts are ongoing to improve our understanding of the pathogenesis of the diseases. Female *Anopheles* mosquito injects MPs into its hosts during a blood meal, and MPs invade the host skin and the hepatocytes without causing any serious symptoms. Symptomatic infections occur only during the erythrocytic stage. In most cases, the host’s innate immunity (for malaria-naïve individuals) and adaptive immunity (for pre-exposed individuals) mount severe attacks and destroy most MPs. It is increasingly understood that MPs have developed several mechanisms to escape from the host’s immune destruction. This review presents recent knowledge on how the host’s immune system destroys invading MPs as well as MPs survival or host immune evasion mechanisms. On the invasion of host cells, MPs release molecules that bind to cell surface receptors to reprogram the host in a way to lose the capacity to destroy them. MPs also hide from the host immune cells by inducing the clustering of both infected and uninfected erythrocytes (rosettes), as well as inducing endothelial activation. We hope this review will inspire more research to provide a complete understanding of malaria biology and promote interventions to eradicate the notorious disease.

## Introduction

Malaria is a major public health concern and is endemic in sub-Saharan Africa and parts of South-Eastern Asia, where over 95% of the 241 million cases and 627,000 malaria-associated deaths were recorded (WHO 2021). Malaria develops when an individual receives a bite from an infected female *Anopheles* mosquito, leading to the injection of malaria parasites (MPs, also known as *Plasmodium* species). Among the species of *Plasmodium* that infect humans, *P. falciparum* is the most notorious, followed by *P. vivax*, with weaker contribution by *P. ovale*, *P. malarae*, and *P. knowlesi* (Okagu et al. 2022)*.* Upon injection into the host, MPs (sporozoites) migrate into the human host’s liver and infect the hepatocytes. They develop into schizonts and release many merozoites into the bloodstream to infect erythrocytes. Most of the pathological effects of malaria infection are accrued to the erythrocytic life cycle stage (Chandley et al. 2023).

Nonetheless, the above situation occurs only when MPs can evade the attack of the host immune system. This is because, on an invasion of the host’s body, the host immune system mounts an immunological response against the parasite (sporozoites stage), leading to the clearance of the invading MPs in most cases (Sierro and Grau 2019). However, as a survival mechanism, MPs have developed several strategies to escape the host immunological attack, starting from the mechanical barrier of the skin to Kupffer cells in the hepatocytes. MPs multiply in the host’s erythrocytes, igniting inflammatory response, oxidative stress, and several hematological and biochemical alterations resulting in complications in untreated malaria. The early stage of malaria infection represents a good strategy for controlling the disease as only a few sporozoites (≤ 200) are injected through mosquito bites compared to over 10,000 merozoites produced in later stages of MPs’ infection in the host (Ménard et al. 2013). Previous reviews, including ours, discussed some biological and hematological responses to malaria infection and hosted immune responses (Akinosoglou et al. 2012; Madrid et al. 2015; Brown et al. 2019; Okagu et al. 2022). Recent findings showed that malaria parasites invade host cells and increase the expression of polymorphic microRNA to induce apoptosis of lymphocytes (Dieng et al. 2020). They also suppress host immune response by inhibiting c-Jun N-terminal kinase phosphorylation in the toll-like receptor 2 signaling pathway of macrophages as mediated by fibrinogen-like protein 2 (Fu et al. 2020). Furthermore, other studies have recently shown that elevated levels of angiogenic and endothelial activation molecules, including vascular endothelial growth factor (VEGF)–A and its receptor, vascular endothelial growth factor receptor 2 (VEGFR2), intercellular adhesion molecule (ICAM)–1, and von Willebrand factor (vWF), and endothelial protein C receptor (EPCR), among others, induce sequestration and evade splenic clearance even in asymptomatic infected persons (Tuikue Ndam et al. 2017; Ukegbu et al. 2020; Frimpong et al. 2021). Acknowledging that malaria parasites can only establish infection upon invasion of the host’s defense (Gaur and Chitnis 2011; Vaughan and Kappe 2017; Agop-Nersesian et al. 2018; Tannous and Ghanem 2018), we aim to provide a brief up-to-date discussion on how the host immune system mounts an immunological attack against MPs and how MPs escape from this attack to cause the disease.

We used keywords and phrases such as “malaria parasite infection,” “host immune system evasion,” “host infection,” “*Plasmodium* species infection,” “malaria parasite survival in hosts,” “rosetting,” and others to retrieve articles published in peer-reviewed journals and indexed in reputable databases such as Web of Science, PubMed, Google scholar publications, and Google search engine. Some recent articles were retrieved from the list of articles citing old publications, while others were from the reference list of recent publications. We first scanned the abstracts for relevance and included papers discussing how host immune responses fight malaria parasites and strategies through which malaria parasites invade host immune attacks.

## Host’s immune response to *Plasmodium* infection

The MPs, in the form of sporozoites, injected into the host’s skin during an infective mosquito bite from a female *Anopheles* mosquito need to reach the liver to continue with the life cycle (Ménard et al. 2013; Venugopal et al. 2020); the detailed life cycle of the parasite within and outside human host has been discussed elsewhere (Ménard et al. 2013; Bucşan and Williamson 2020; Venugopal et al. 2020). The host immune response to the malaria parasite invasion on the skin, the liver, and erythrocytes was briefly discussed below, highlighting how MPs are destroyed before they establish symptomatic infection in immunocompetent hosts.

### Immune responses to the presence of sporozoites in the skin

In a healthy individual (immunized or not), skin damage inflicted by a probing bite of a mosquito (even a sterile one) stimulates the degranulation of mast cells, leading to recruitments of immune cells to the dermis and epidermis (Demeure et al. 2005; Voss et al. 2021). Neutrophils and, later, monocytes, are the first circulating immune cells recruited after a mosquito’s bite. Their levels are sustained long if the mosquito’s saliva contains sporozoites (Mac-Daniel et al. 2014; Hopp and Sinnis 2015). Although neutrophils can phagocytose sporozoites leading to their imminent death, the killing of sporozoites by neutrophils in the skin may not be very significant, especially in malaria naïve individuals, as demonstrated by the lack of correlation between the number of naïve mice’s neutrophils and the number of parasites developing in their livers after sporozoites’ intradermal inoculation (Mac-Daniel et al. 2014; Hopp and Sinnis 2015). Moreover, the expression and secretion of agaphelin through mosquito saliva are heightened after *P. falciparum*’s mosquito infection; this protein inhibits human neutrophils’ activities, probably diminishing the associated protection (Waisberg et al. 2014; Aitken et al. 2018). Also, some sporozoites can escape phagocytosis through cell traversal mechanism and also probably by outpacing of host immune cells (sporozoites glide through the skin at 1–2 μm/s which is considerably higher than 0.1 μm/s of the host’s immune cells) (Mac-Daniel et al. 2014; Hopp and Sinnis 2015). Thus, the parasite can even exit the skin before infiltration of neutrophils (Hopp and Sinnis 2015) and proceed to infect the liver, especially in malaria-naïve individuals. Other mechanisms through which sporozoites bypass immune responses are discussed in detail later. Apart from mast cells’ degranulation, the injection of sporozoites increases the motility of skin regulatory T cells and dendritic cells (DCs) (da Silva et al. 2012), causing the DCs (which recognize pathogen-associated molecular patterns, PAMPs) to phagocytose the invading MPs (sporozoites). In this process, naive CD4^+^ and CD8^+^ T cells are also activated by presenting the sporozoites’ antigens on major histocompatibility complex (MHC) molecules (Osii et al. 2020)**.** CD4^+^ T cells (which recognize antigens on MHC class II molecules of antigen-presenting cells) produce pro‐inflammatory responses such as upregulated expression of interleukin (IL)-12, interferon-gamma (IFN-γ), and inducible nitric oxide synthase (iNOS) (Donovan et al. 2007; Osii et al. 2020) to destroy MPs. They also help in CD8^+^ T cell activation and humoral immunity (production of antigen-specific antibodies by B-cells) (Osii et al. 2020). CD8^+^ T cells (which recognize antigens on DCs’ MHC class I molecules), on the other hand, attack pathogens and/or pathogen-infected cells through secretion of cytokines [e.g., IFN-γ and tumor necrosis factor-alpha (TNF-α)] (Chakravarty et al. 2007; Villarino and Schmidt 2013), the release of cytotoxic granules (Junqueira et al. 2018; Osii et al. 2020), or by activation of Fas/FasL-mediated caspase cascade (Imai et al. 2015). However, the high frequency of clinical malaria, especially among unvaccinated malaria-naïve infants (Natama et al. 2018) suggests that phagocytosis by DCs and first-time activation of CD4^+^ and CD8^+^ T cells are not fast or efficient enough to protect against the parasites’ challenge**.** Adults residing in malaria endemic zones are naturally exposed to several infections by MPs via mosquito bites, leading to the acquisition of clinical immunity against blood-stage malaria—see previous reviews for further details—(Doolan et al. 2009; Barry and Hansen 2016; Frimpong et al. 2020; Gonzales et al. 2020; O’Flaherty et al. 2022). This is due to the activities of mainly peptide-specific CD8^+^ T and CD4^+^ T helper cells acquired from previous infections (Sedegah et al. 1992; Kurup et al. 2019). Sterile protection, however, is not impacted through natural exposure (Tran et al. 2013; Osii et al. 2020), probably due to the high genetic variability of pre-erythrocytic antigens in high transmission areas (Barry et al. 2009; Tran et al. 2013) or due to a small number of sporozoites inoculated per bite, leading to incomplete adaptive response towards the pathogen (Tran et al. 2013; Hopp and Sinnis 2015). Thus, some of the injected *Plasmodium* sporozoites, unaffected by the elevated dermal immune response acquired from previous bites, exit the dermis and move into the bloodstream for transfer to the liver or into the lymphatic circulation, although some can remain at the inoculation site, and transform to the exo-erythrocytic stage (merozoites) (Gueirard et al. 2010). In individuals immunized using sporozoites-based vaccines, such as, circumsporozoite protein-specific monoclonal antibodies, immunity to clinical malaria, and sterile protection involving humoral and CD8^+^ T cell-based responses are achievable at high antibody titer (Olotu et al. 2013; Hopp and Sinnis 2015; Livingstone et al. 2021). The antibodies act through different mechanisms, including reducing the number of sporozoites injected during bites or reducing the parasites’ motility in the skin (Kebaier et al. 2009; Flores-Garcia et al. 2018).

In the blood of an adult previously and severally infected by (usually in endemic malaria zone) or vaccinated with sporozoite-based vaccines, IgG1 and IgG3 (which readily binds to Fcγ receptors, FcγR) dominate the antibody-mediated responses (Hoffman et al. 1986; Chua et al. 2021; Feng et al. 2021). The circumsporozoite protein (CSP) of sporozoites that exited the dermis is recognized and bound by these antibodies, neutralizing the proteins needed for cell traversal and invasion (Belachew 2018) and opsonizing the sporozoites for phagocytosis and destruction mainly by neutrophils (Feng et al. 2021). This hence reduces the number of sporozoites that can infect the liver.

### Immune responses to liver-stage Plasmodium infections

**Fig. 1:**
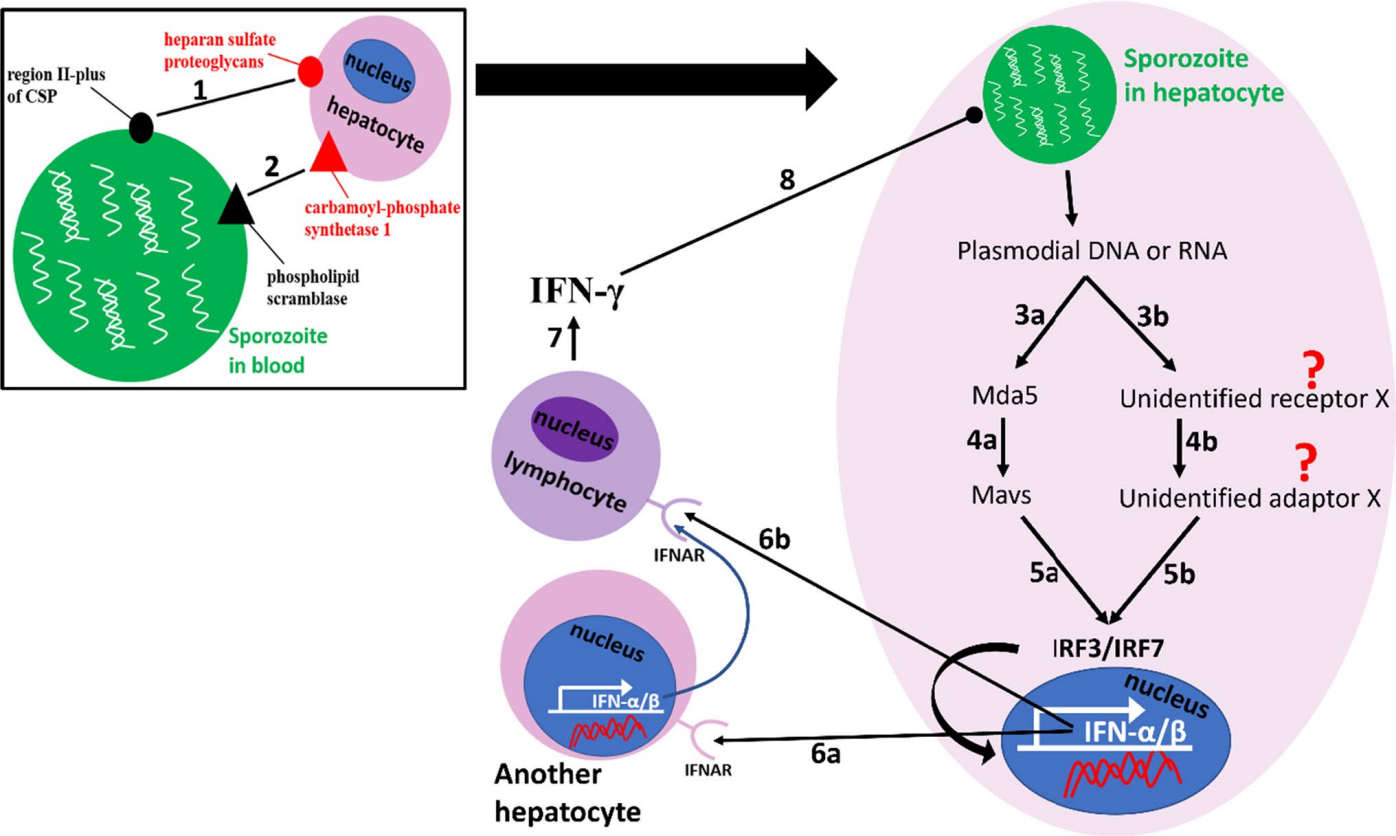
Immune system recognition of and responses to liver-stage plasmodial infection: the region II-plus region of CSP and/or the phospholipid scramblase of the sporozoite can, respectively, bind to the heparan sulfate proteoglycans and/or the carbamoyl-phosphate synthetase on a hepatocyte (1 and 2), followed by invagination and entry. In the infected hepatocyte, the plasmodial RNA or DNA can be recognized by MDA5 or another unknown receptor(s) (3a and 3b), which can trigger the adaptor MAVS or other unidentified adaptor(s) (4a and 4b). The adaptors, in turn, trigger IRF3 and IRF7 (5a and 5b) to activate transcription and expression of IFN-α and IFN-β (type I IFNs). Type I IFNs bind to IFNAR on other hepatocytes to induce the expression of more type I IFNs (6a) or bind to the IFNAR on lymphocytes (especially NKT cells) (6b), which expresses IFN-γ (7) for inhibition of sporozoites multiplication (8)

There are various ways through which sporozoites recognize and gain access to liver cells. The sporozoite can use the region II-plus of CSP to bind to heparan sulfate proteoglycans projecting from endothelial cells, Kupffer cells (KCs), and hepatocytes (Patarroyo et al. 2017; Shabani et al. 2017) or bind to CD68 receptors on KCs (Cha et al. 2015) or use its phospholipid scramblase to interact with the carbamoyl-phosphate synthetase 1 on hepatocytes’ membranes (Cha et al. 2021). These successful interactions are followed by the release of invasion ligands (e.g., thrombospondin-related adhesive protein and rhoptries-associated proteins) from the sporozoite’s micronemes and rhoptries, formation of tight junction, and engagement of an actomyosin motor complex, which leads to invagination of host cell’s plasma membrane, entry of sporozoites and formation of parasitophorous vacuole membrane (PVM) (Vaughan and Kappe 2017), similar to what happens when merozoites infect erythrocytes (Vaughan and Kappe 2017; Okagu et al. 2022). Once inside the host’s cell, the parasite can traverse through adjacent cells until a final cell establishes an infection. Although the liver stage of infection is asymptomatic, the host does not remain passive. The host responds to the presence of the parasites by expressing interferons (type I and II IFNs) (Liehl et al. 2014; Miller et al. 2014), and both interferon types play different roles in the recognition and elimination of pathogens. Different researchers, however, have different opinions on the roles and indispensability of specific sensors (receptors) and adaptors for initiating type I IFN response. According to Liehl et al. (Liehl et al. 2014), an infected hepatocyte senses the *Plasmodium* RNA (a PAMP) through its melanoma differentiation-associated gene 5 (MDA5) [an example of cytoplasmic pattern recognition receptor (PRR)], which triggers mitochondrial antiviral signaling protein (MAVS) [another PRR]. MAVS then triggers the indispensable interferon regulatory factors, IRF3 and IRF7, to activate transcription and expression of IFN-α and IFN-β (type I IFNs). These two IFNs then bind to IFNAR (IFN-α/β receptors) on hepatocytes and on leukocytes to activate interferon-stimulated genes (ISGs) (see Fig. 1). Miller et al. (Miller et al. 2014), however, observed that deletion of IRF7 and the adapters MDA5, MAVs, toll-like receptors (TLRs), stimulator of interferon genes (STING), etc. did not adversely hamper type I IFN signaling, showing that the indispensable IRF3 might be triggered via an unidentified pathway. Moreover, the induction of type I IFNs in bone marrow cells stimulated by malarial genome-derived AT-rich stem-loop DNA and *P. falciparum* genomic DNA was not hampered by MAVs, MDA5, retinoic inducible gene-I, and TLR deletions, but were strictly dependent on STING, tank-binding kinase 1 (TBK1), and IRF3/7 (Sharma et al. 2011). These differences might be due to experimental models and different antigens (sporozoites, plasmodial genomic DNA, AT-rich stem-loop DNA, plasmodial RNA, etc.) used in different studies.

Many cells in the liver express the INFAR. Still, only hepatocytes seem indispensable for recognizing pathogens as the specific deletion of INFAR on hepatocytes, but not the specific deletion on macrophages and neutrophils, hampered the induction of ISGs (Liehl et al. 2014). This makes sense since liver-stage infection begins when sporozoites multiply in hepatocytes. Although the hepatocytes are essential for initiating type I IFN response, other cells and perhaps other signaling pathways may be necessary to eliminate pathogens. The observation evidences that type I IFN response led to reduced liver stage infection in vivo but not ex vivo (using mouse hepatocytes) (Liehl et al. 2014). This is also supported by the report of Miller et al. (Miller et al. 2014), who observed that both IFN-γ (a type II IFN produced only by activated lymphocytes) and IFN-β (a type I IFN) mediated the liver stage immune responses in both C57BL/6 and BALB/cJ wild-type mice strain injected with attenuated *Plasmodium yoelii* sporozoites (which can complete liver-stage cycle but cannot progress to the blood stage).

Moreover, liver stage infection was significantly reduced in wild-type C57BL/6 mice compared to an IFN-γ knockout (IFN-γ^−/−^) mice (Miller et al. 2014). Ifn-γ-stimulated hepatocytes have been demonstrated to inhibit sporozoites multiplication by initiating noncanonical autophagy (Boonhok et al. 2016), emphasizing the contribution of type II IFN and not only type I IFN in hepatic stage immune responses. During the liver stage infection, the type I IFN-mediated response results in the recruitment of lymphocytes, especially natural killer T (NKT) cells, to eliminate sporozoites, especially during secondary sporozoites infection. It should be noted, however, that the important roles of NKT cells are diminished in the livers of infected IFNAR^−/−^ mice, emphasizing the importance of type I IFN signaling in the enrichment or recruitment of NKT cells (Miller et al. 2014). We thus conclude that type I IFN signaling, initiated via various pathways (which should be clarified in future studies), is necessary for liver-stage pathogen recognition and lymphocyte enrichment, while type II IFN response is important for pathogen elimination.

### Immune responses to blood-stage Plasmodium infection

**Fig. 2:**
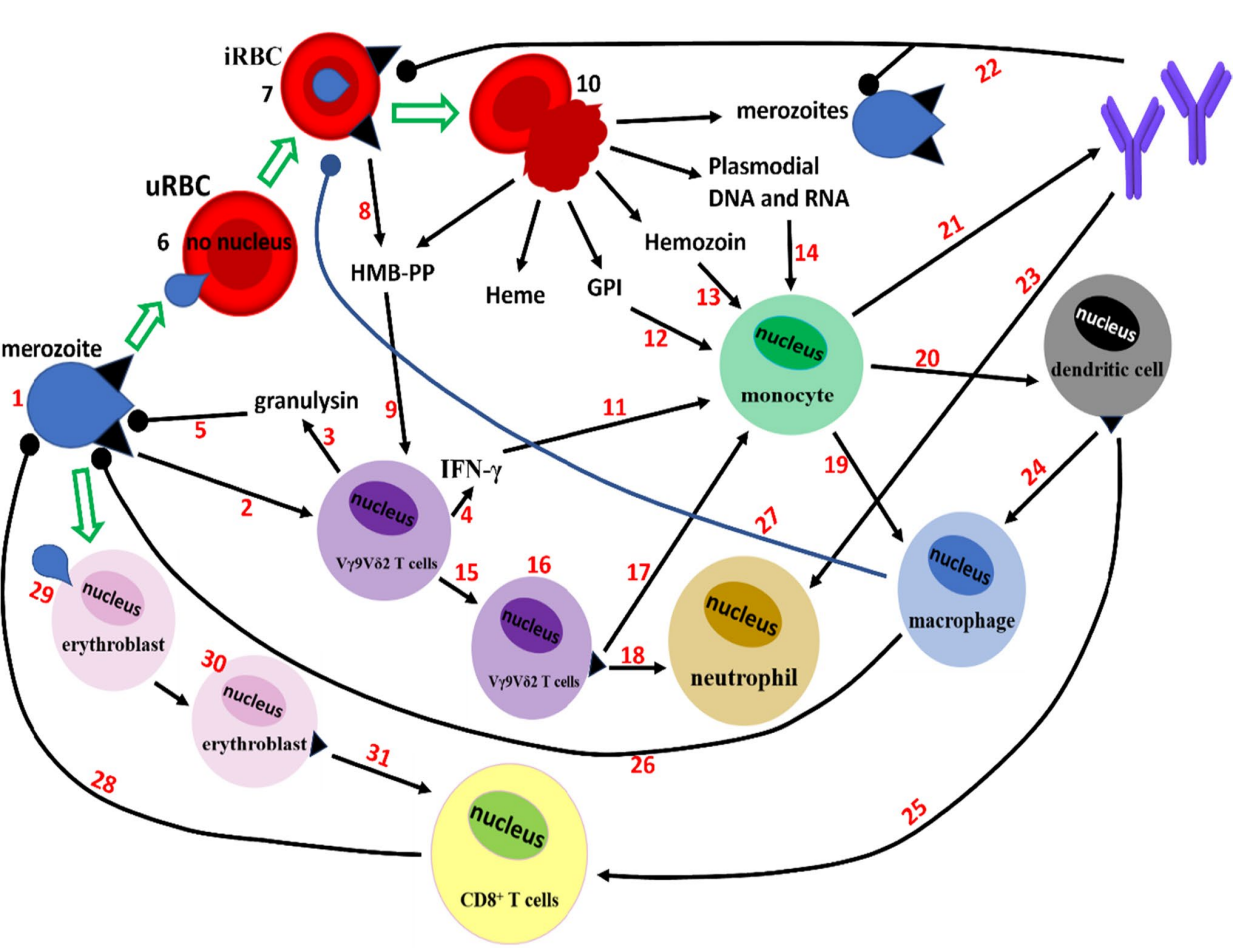
Immune system recognition of and responses to blood-stage plasmodial infection: merozoite (1), through an unclear mechanism, can be recognized (2) by Vγ9Vδ2 T cells, which can release granulysin (3) and IFN-γ (4). The granulysin can inhibit merozoites (5) but requires cell-to-cell contact. Uninhibited merozoites can invade (6) RBCs to produce iRBCs (7), which expresses the parasite’s proteins on its surface and also releases phosphoantigens (8) into the extracellular environment. These phosphoantigens can also activate (9) Vγ9Vδ2 T cells. iRBCs can burst (10) to release new merozoites and many metabolites, activating other immune cells. Aside from being activated by IFN-γ released by Vγ9Vδ2 T cells (11), monocytes can be activated by GPI, hemozoin, and plasmodial DNA and RNA (12–14). Vγ9Vδ2 T cells can also process (15) and present (16) the parasite’s proteins to monocytes (17) and neutrophils (18). Activated monocytes can differentiate into macrophages (19) or dendritic cells (20) and can also secrete B-cell activation factors (21) for the development of specific antibodies. The antibodies can bind to and opsonize (22) merozoite and iRBCs, and interact (23) with neutrophils to destroy opsonized merozoite and iRBCs. Dendritic cells can process and present antigens via MHC molecules to macrophages (24) and CD8^+^ T cells (25). The activated macrophages can phagocytose and destroy merozoites (26) and iRBCs (27) expressing parasite proteins on their surfaces. Activated CD8^+^ T cells produce cytokines that attack pathogens (28). Merozoites, in some cases, can attack erythroblasts (29), which will process and present the parasite’s antigens via MHC class I molecules to CD8^+^ T cells, which respond as already described. uRBCs = uninfected red blood cells

Through various mechanisms (discussed later), especially in unvaccinated malaria-naïve individuals, the sporozoites can still escape recognition and destruction by the liver-stage immune response, grow, and reproduce to form merozoites-containing schizonts (Fig. 2). The ruptures of an infected hepatocyte and a schizont (which ends the pre/exo-erythrocytic stage) release thousands of merozoites (Ménard et al. 2013; Venugopal et al. 2020), whose surface proteome differs from that of sporozoites, making them unsusceptible to sporozoites-specific immune responses (Bucşan and Williamson 2020). Costa et al. (Costa et al. 2011) demonstrated that these extraerythrocytic merozoites, via a TCR-dependent manner, can activate the (non-MHC-restricted) Vγ9Vδ2 T cells, a subset of γδ T cells to release merozoites-sensitive cytotoxins (especially granulysin) to inhibit the parasites, with this inhibition requiring a cell-to-cell contact (between Vγ9Vδ2 T cells and the parasite). This recognition of merozoites by Vγ9Vδ2 T cells should represent an important step to abrogating RBC infection (and blood-stage malaria) and may have the potential for the development of merozoite-specific vaccines. However, the study neither confirmed the merozoite-derived metabolite(s) (which could be phosphoantigens) that is recognizable by the Vγ9Vδ2T cells nor confirmed whether proximity between merozoites and Vγ9Vδ2 T cells is necessary for the Vγ9Vδ2 T cell activation. Activated Vγ9Vδ2 T cells can also present antigens at lymph nodes to activate other immune cells, including monocytes, neutrophils, and NK cells (Bucşan and Williamson 2020; Eberl 2020; Herrmann et al. 2020). Undetected/undestroyed merozoites, using their surface proteome, infect erythrocytes to form PVMs; newly formed-merozoites are released upon rupture of infected red blood cells (iRBCs), and clinical symptoms accompany this—see previous review (Okagu et al. 2022) for details. This RBC infection-merozoites release cycle continues until parasites are cleared by the host’s immune system, by chemotherapy, or by the host’s death (Bucşan and Williamson 2020). During early merozoites’ multiplication in iRBCs, the parasites are protected from the immune response as RBCs do not express MHC molecules. They cannot stimulate cytotoxic T cells (Bucşan and Williamson 2020), although parasite proteins and other metabolites are trafficked to the iRBCs’ surface and the extracellular microenvironment. In as much as DCs have been demonstrated to phagocytose iRBCs, activation, and maturation of the DCs after exposure to iRBCs is dose-dependent, and high iRBCs:DCs ratio leads to DCs’ apoptosis (Elliott et al. 2007; Bucşan and Williamson 2020), making early recognition of blood-stage (exoerythrocytic) malaria by DCs unreliable. Apart from the infection of mature RBCs, it has also been demonstrated that RBC precursors are susceptible to malaria parasite infection (Tamez et al. 2009; Imai et al. 2013). Infected erythroblasts expressing MHC class 1 molecule can activate CD8^+^ T cells to bring about IFN-γ expression, cytotoxicity, etc. (Imai et al. 2013). However, since the parasites prefer to infect later stages of erythroid cells (Tamez et al. 2009; Imai et al. 2013), we suppose that recognizing blood-stage parasites after RBC infection is paramount. Early recognition of blood-stage infection is efficiently done by the Vγ9Vδ2T cells in a TCR-dependent, but not proximity-dependent manner, as the phosphoantigens (intermediate metabolites of the parasite’s DOXP pathway) released by iRBCs readily activate Vγ9Vδ2T cells, without requiring cell-to-cell contact (Guenot et al. 2015). iRBCs can also burst to release new merozoites and parasite-generated metabolites such as hemozoin, heme, (E)-4-hydroxy-3-methyl-but-2-enyl pyrophosphate (HMB-PP), and glycosylphosphatidylinositol (GPI). Apart from being activated by cytokines (e.g., IFN-γ) released by Vγ9Vδ2T cells, monocytes (which can differentiate into macrophages and DCs) can also be activated by hemozoin (often coated by plasmodial DNA and RNA) and GPI (which can also activate NKT cells), via toll-like and lectin receptors, leading to phagocytosis, antigen presentation, and secretion of B cell activation factor (Kumsiri et al. 2010; Gazzinelli et al. 2014; Bucşan and Williamson 2020). Activated neutrophils can also respond to infections through complement-dependent phagocytosis, production of reactive oxygen species (ROS), and formation of neutrophil extracellular traps (Bucşan and Williamson 2020). After these series of T and B cell activation, parasites’ proteins displayed on merozoites and/or iRBCs also become recognizable by specific antibodies for opsonization and destruction (Meinderts et al. 2017; Okagu et al. 2022). Also, iRBCs expressing parasites’ proteins have altered membrane flexibility and cytoplasm viscosity and are thus trapped in the spleen and cleared by phagocytes, especially macrophages (Fig. 2) (Meinderts et al. 2017; Depond et al. 2020). These responses of the host’s immune system sometimes contribute to clinical symptoms of malaria, such as fever and anemia, as discussed elsewhere (Sharma et al. 2011; Okagu et al. 2022).

## Malaria parasite survival starts from the mosquito host — how malaria parasites evade mosquito’s immune response

Mosquitoes are the primary host of *Plasmodium* parasites as well as their transmission vector (Okagu et al. 2022). It is fascinating to highlight how *Plasmodium* parasites survive and escape their primary host’s bullet (mosquito immune response). Mosquito infections with *P. falciparum* involve cascades of events immediately after a blood meal (via mosquito bites) containing the *Plasmodium* gametocytes (Venugopal et al. 2020). In the midgut lumen of mosquitoes, the gametocytes differentiate and mature into gametes. The gametes, after fertilization, form the zygotes, which then mature into an ookinete (Nakayama et al. 2021). The first mosquito’s innate immunity is the physical peritrophic membrane barrier, which should prevent the migration of the ookinete to the basal lamina. However, the ookinetes secrete degradative enzymes, such as chitinase, that disintegrate the physical peritrophic membrane barrier (Chandley et al. 2023).

More so, in the midgut, the ookinete is attacked by a myriad of midgut proteases; however, the ookinetes express surface protein such as P25, P28, and P47 that evade those proteases (Molina-Cruz et al. 2015). Very popular in recent studies is the P47 encoded by the Pf47 gene, known to disrupt the compliment-like immune responses of mosquitoes as well as inhibit the JNK pathway-mediate apoptosis of ookinete (Molina-Cruz et al. 2020). In a recent study, a lock and key model was proposed with strong evidence for the action of P47 protein on its receptor P47Rec (Pfs47 receptor- the lock) — in the midgut (Molina-Cruz et al. 2023). Silencing/downregulation of the P47Rec reduced the parasite infection (Molina-Cruz et al. 2020). Other well-studied ookinete surface proteins are the guanylate cyclase β (GCβ) — which mediate the ookinete migration, and putative secreted ookinete surface protein (PSOP25) — which promotes the maturation of ookinete (Nakayama et al. 2021).

The parasite ookinete successfully migrates from the midgut to basal lamina, where there are challenged heavily by the myriad of mosquitos’ immunity, theoretically strong enough to reduce the ookinete number by a thousand folds. As a survival response, the few ookinetes left differentiate into oocyst, which is covered by a hemolymph-containing capsule resistant to mosquito immunity (Singh et al. 2021). The oocyst within the capsules then differentiates into thousands of sporozoites. In very recent studies, several proteins or genes have been identified for their functional role in promoting the evasion of mosquito immunity by malaria parasites (Keleta et al. 2021).

The *Plasmodium* infection of the Mosquito Midgut Screen 43 (PIMMS43) is becoming popular in recent studies and is known for its activity in promoting the evasion of mosquito complement-like response. This protein was reported to be expressed on the surface of both ookinete and sporozoites (Chandley et al. 2023). A recent study reported that the downregulation of PIMMS43 or its antibody inhibition caused a significant decrease in mosquito infection with the *Plasmodium* parasite. Therefore, it suggested PIMMS43 as a potential drug target for malaria (even before the parasite infects humans) (Ukegbu et al. 2020).

In another study, the circumsporozoite protein (CSP) was reported as another important protein that fosters the evasion of mosquito immunity by *Plasmodium* oocysts. The researchers discovered that parasite oocysts with a knockdown expression of CSP_mut_, on infection with mosquito-induced hemocyte nitration, mediate by NADPH oxidase 5 (NOX5), which then fosters the melanization of matured oocysts, upregulated expression of hemocyte TEP1 and a corresponding distorted release of sporozoites (Zhu et al. 2022). Furthermore, it was recently reported that the post-translational modification with glutaminyl cyclase of some parasite proteins, such as CSP, promotes their evasion. When the CSP’s glutaminyl cyclase target (glutamine) was subjected to mutation, a corresponding melanization of the sporozoites was observed. Hence the glutaminyl cyclase can be another druggable target (Kolli et al. 2022). Several other proteins, such as TRAP, MSP, and others, have been reported to play critical roles in parasite evasions (Nakayama et al. 2021). These findings from recent studies have opened more possibilities to discover new drugs, especially from natural products, and combat antimalarial resistance (Okeke et al. 2021; Ezeorba et al. 2022; Chukwuma et al. 2023).

## Evasion of human host immune response by malaria parasites

Malaria infections are very complex in their etiology and mode of infection. The condition caused by *P. falciparum* or *P*. *vivax* has myriad symptoms as the parasite gains entries and infects the erythrocytes (Cowman et al. 2016). Moreover, several events occur at the pre-erythrocytic stages. Sporozoites originating from the female *Anopheles* mosquito vector during a blood meal are injected into the human host and liver cells (Venugopal et al. 2020). Despite the asymptomatic nature of the pre-erythrocytic and hepatic stages, a diverse set of immunological reactions in response to the parasites usually occurs (Abuga et al. 2021), as discussed above. However, *P. falciparum* has evolutionarily gained several evasion mechanisms, ensuring they still securely inflict malaria infection even in immune-competent patients, despite the myriads of immunological response (Gomes et al. 2016; Rénia and Goh 2016). Understanding the mechanism of immunological evasion by malaria parasites would better inform innovations of effective therapies and discoveries of potent vaccine candidates (Tannous and Ghanem 2018; Pollard and Bijker 2020; Mandala et al. 2021). Several studies have successfully elucidated some of these evasion mechanisms over the past decades, summarized in many previous reviews (Zheng et al. 2014; Gomes et al. 2016; Rénia & Goh 2016; Tannous & Ghanem 2018). Therefore, this session gives an up-to-date overview of the immunological evasion mechanisms of malaria parasites by summarizing what has been previously known and recently discovered.

### Evasion in the human host starts from the skin as a mechanical barrier

The skin is the first port of call to resist malaria infection or parasitic entry from a mosquito bite. Despite the innate defensive role of the skin, some sporozoite stills beat the skin defense and gain entry into the body (Belachew 2018; Kalia et al. 2021). The enormous number of sporozoites (about 100 to 200) released into the human skin on a single mosquito bite presents an overwhelming pressure for the human skin to defend against, although its ability to reduce the number that gains entry drastically is very remarkable (Hopp and Sinnis 2015). It has been reported that sporozoites have evolved with adequate motility apparatus and cell transversal properties, favoring their evasion of the skin barrier (Gomes et al. 2016). Mechanical proteins such as “sporozoites microneme protein essential for cell traversal” (SPECT-1 and 2) and perforin-like protein 1 (PLP1) have conferred resistance and provide easy motility of *Plasmodium* through the skin barrier (Ejigiri and Sinnis 2009; Guerra and Carruthers 2017). A study by Patarroyo et al. (2011) reported that *Plasmodium* with downregulated or deficient SPECT 1 and 2 or PLP1 were blocked in the dermis layer of the skin and, after that, ingested by phagocytes. Furthermore, it was discovered that these mechanical proteins also facilitate the quick migration of sporozoites into the liver (Gomes et al. 2016). Another mechanical protein, TRAP (thrombospondin-related anonymous protein), has been found on the surface of sporozoite's micronemes and implicated with facilitating the gliding motility and binding of sporozoites to sulfated glycoconjugate motifs for hepatocyte recognition, binding, and entry (Wilson et al. 2016).

### Evasion of the immune defense in the liver cells to establish the hepatocytic infection

After sporozoites gain entry into the blood from the skin, they quickly move through the circulatory system until they get attached to the sinusoid cavity of the liver (Frischknecht and Matuschewski 2017). Due to the immunomodulatory nature of the liver to resist extensive inflammation, there are no pathological symptoms experienced in establishing hepatocytic infection by *P. falciparum* (Gomes et al. 2016). Recent studies have shown that despite the immune regulatory activities in the liver, myriad immunological defenses are posed against parasitic infection; however, sporozoites have evolved with several evasion mechanisms (Tran and Crompton 2020). Some immune responses against *Plasmodium* activate the IFNs pathway as the *Plasmodium* RNA binds to an MDA5 receptor, a cytosolic pattern recognition receptor in the liver (Gomes et al. 2016). Other activities are mediated by NK cells, natural killer T cells, γδT cells, and hepcidin, which inhibit the parasitic growth in the liver (Burrack et al. 2019). The two major routes through which parasitic *Plasmodium* secures their infections in the liver regardless of the immunological response are the modulation of the Kupffer cells and hepatocytes on entry and liver cells, respectively (Gomes et al. 2016).

The Kupffer and endothelial cells are phagocytic cells that line as barriers on the hepatocytes’ surface (outer sinusoidal layer) (Bertolino and Bowen 2015). Several studies have reported that *Plasmodium* modulates Kupffer and endothelial cells’ activities to gain entry into the hepatocytes (Tweedell et al. 2018). A study on mice recently reported the lowered expression of Th1 cytokines [TNF-α, IL-6, and monocyte chemoattractant protein-1 (MCP-1)], while upregulation of Th2 cytokines (IL-10) was observed as sporozoite enters the liver. It is well-known that the sporozoites usually express a CSP, which is adapted to attach to the sulfated heparin sulfate proteoglycans (HSPGs), usually on the surface of the hepatocyte (Deslyper et al. 2019). Moreover, studies have implicated the CSP to interact with LRP-1 (low-density lipoprotein receptor-related protein) and other proteoglycans. The CSP-LRP-1 interactions fostered the increase in cAMP/EPAC, which concomitantly hinders the formation of ROS against the parasite (Ikarashi et al. 2013). Other studies have reported the parasite’s activities in interfering with the antigen presentation capacity of the Kupffer cells, such as downregulating the expression of MHC class 1 and IL-12, producing overall immunosuppression of the hepato-microenvironment (Osii et al. 2020). Moreover, in critical conditions, the parasites foster the apoptosis and inactivation of the Kupffer cells (Ozarslan et al. 2019).

On a successful entry into the hepatocyte, after conquering the natural sinusoidal immunological barriers, the sporozoites adopt the cholesterol uptake pathway, unique and different from other parasitic attacks, to invade the hepatocyte (Deroost et al. 2016). In the hepatocyte, the sporozoites release their CSP. Studies have shown that the sporozoites’ CSP modulates inflammatory response by downregulating the host NF-kB signaling pathway and upregulating the heme oxygenase-1 (HO-1) (Belachew 2018). Moreover, the mTOR pathway, which regulates cell survival, proliferation, anabolism, autophagy, and cell growth, is also altered by the activities of the parasite CSP (Dimasuay et al. 2017; Rashidi et al. 2021). After establishing infection, the sporozoite in the liver is covered by PVM, which isolates and protects the host cells from endocytic and lysosomal activities in the cytoplasm of hepatocytes (Gomes et al. 2016; Agop-Nersesian et al. 2017, 2018).

Moreover, the PVM prevents the cells from apoptosis and selective autophagy, ensuring survival. The sporozoite in the liver is transformed into the merozoites on exit from the liver to infect the erythrocytes (Agop-Nersesian et al. 2018). On budding off the liver cells, the merozoites are enveloped in a membrane known as merosomes, which protect the parasite from phagocytic attack and other forms of immune response (Niklaus et al. 2019). Hence, the parasite successfully exits the hepatocytes into the blood for infection of erythrocytes.

### Merozoites infect erythrocytes and evade immunological attacks to establish clinical symptoms

In the blood, the merozoites released from the liver utilize several complex proteins expressed on their surfaces to infect (or gain entry into) RBCs. Studies have identified the merozoite surface proteins (MSP-1) and other erythrocyte binding-like (EBL) proteins to facilitate the parasite attachment to the surface membrane of erythrocytes through a protruded GPI anchor (Nosjean et al. 1997). It was also recently discovered that MSP and EBL proteins of merozoites are highly polymorphic and are expressed as several alleles or copies in the parasite’s genome (Gomes et al. 2016). Hence, these surface proteins delude the immunological defenses against the parasites. Apart from the GPI on the surfaces of the erythrocytes, some individuals have been discovered to have Duffy antigen receptor chemokines (DARC) on their erythrocytes (Golassa et al. 2020). The DARC has been reported to foster merozoites to gain entry into the erythrocytes; hence, individuals with DARC are more susceptible to progressive malaria (Miri-Moghaddam et al. 2014).

Once the merozoites infect erythrocytes, they develop into ring-shaped trophozoites, which rapidly undergo schizogony — a type of multiple fusion from one cell, producing six schizonts, then 32 daughter clones, which then results in erythrocytes’ apoptosis and release of the immature clones into the bloodstream (Belachew 2018). The schizogony phenomenon fosters the parasite’s rapid spread, infecting many erythrocytes in the circulatory system (Rund et al. 2016). A vast array of immunological responses to the activities of merozoites and trophozoites have been reported ranging from antibody identification of infected RBCs (iRBCs) and opsonization/phagocytosis by macrophages (Bucşan and Williamson 2020); to T cells, activities fostering the secretion of pro-inflammatory cytokines (IFN-γ and TNF-α) and activating specific B-cell clones (for specific antibody production) (He et al. 2020); and to natural killer cells, γδT cells, and host-microbiota with other salient immunological defensive functions as described in the previous section (Vijayan et al. 2021).

Malaria parasites have evolved with several mechanisms to evade the immunological defenses in the circulatory system. Moreover, iRBCs typically marked for destruction in the spleen are prevented as well as the activities of complement are inhibited (Mubaraki et al. 2016). Usually, infected and normal erythrocytes are not recognized by CD8^+^ T cells because they do not express MHC-1 on their surfaces, hence, of survival advantage to the parasite (Imai et al. 2015). Conversely, the parasite evades clearance by forming a rosette — a cluster and masking infected RBC by uninfected RBC. The phenomenon of rosette was reported to be more common in blood type A than in blood type O. Hence, individuals with blood type A are more predisposed to be down with severe malaria than other individuals (Moll et al. 2015).

In the last decade, some proteins were identified with sequestration and adherence of iRBCs to microvasculature/endothelia of the different organs to prevent their immunological clearance (Lee et al. 2019). Some of these proteins, especially those mediated by *P. falciparum* erythrocyte membrane protein 1 (*PfEMP-1)*, *Var*, *Rifin*, and *Stevor* multigene families, also facilitate the formation of rosette clusters and have been implicated in other pathological challenges such as malaria anemia, placental malaria in pregnant women, and cerebral anemia (Abdi et al. 2016; Wahlgren and Goel 2017; Sakoguchi et al. 2021; Chew et al. 2022; Okagu et al. 2022). These sequestration/adherence proteins secreted in iRBCs cause the formation of knobs on the surface cell membrane of iRBCs (Fairhurst et al. 2012). The knobs then facilitate the adhesion to endothelium and microvasculature by the solid affinity for a couple of endothelia receptors expressed in different organs such as endothelial protein C receptor (EPCR), chondroitin sulfate A (CSA), intercellular adhesion molecules (ICAMs), and CD36 (Lee et al. 2019; Venugopal et al. 2020). Summarily, in the blood, the parasites first evade the immune response by polymorphic receptors and second through their intense sequestration and adhesion to endothelium. Finally, its rapid and schizogonic replication pattern resists their clearance.

## New mechanistic findings on malaria parasites evasion in human

The immunological evasion by malaria parasites is very complex, and studies have yet to unravel the underlying mechanisms exhaustively. More in-depth knowledge of the mode and means of immunological invasion will provide a more information-driven treatment and vaccine discovery approach (Tan et al. 2018). Here, we summarize the contributions to existing knowledge by several recent studies on mechanisms via which MPs escape from the host’s immune response (Table 1).

**Table 1:** A summarized update on the current understanding of malaria parasite immunological evasion mechanisms

*Plasmodium* species	Secreted protein or gene for evasion	Accession number	Specific immunological factors for evasion	Stage of parasitic attack	Mode of studies	Reference
*Plasmodium berghei NK65*	extracellular vesicles proteins (histamine-releasing factor (HRF) and the elongation factor 1α (EF-1α)	HRF—XP_034422313.1	Inhibits T-cells by dephosphorylating PLCγ1, Akt, and ERK molecules of the T-cells signaling cascade	Blood stage	In vitro and In vivo	(Demarta-Gatsi et al. 2019)
*Plasmodium falciparum*	Acquired host plasma plasminogen is activated by urokinase-type and tissue-type plasminogen activators into serine protease plasmin	NP_776572.1	Degradation of fibrin and C3 and C5 complement factors	Intra-erythrocytic stages	In vitro	(Reiss et al. 2021)
*Plasmodium falciparum*	Infected Erythrocytes opsonized by specific PfEMP1 immunoglobulin (IGG)	PFD37_1200600	Classical complement pathway	Intra-erythrocytic stages	In vitro	(Larsen et al. 2019)
*Plasmodium falciparum*	Glutaminyl cyclase of sporozoite	PKC49730.1	Promote evasion of recognition by mosquito immune system and hemocoel	Invertebrate mosquito host (sporozoites)	In vivo	(Kolli et al. 2021)
*Plasmodium falciparum*	miRNA (miR-16-5p, miR-15a-5p, and miR-181c-5p)	NR_031817.1, NR_029485.1 KT921554.1	Lymphocyte cell death	Intra-erythrocytic stages	In vivo	(Dieng et al. 2020)
*P. falciparum* and *P. berghei*	PIMMS43 (*Plasmodium* infection of the mosquito midgut screen 43)	PF3D7_0620000	Mosquito Complement-like Response upon Midgut Traversal and required for oocytes maturation and sporozoites development	Invertebrate mosquito host (sporozoites)	In vitro and in vivo	(Ukegbu et al. 2020)
*Plasmodium falciparum*	Shed, erythrocyte *binding* antigen of 175 kDa (EBA-175)	AAZ65998.1	RBCs clustering through binding of glycophorin A (GpA)	Intra-erythrocytic stages	In vitro	(Paing et al. 2018)
*Plasmodium vivax or Plasmodium falciparum*	Induces CD4^+^Foxp3^+^CD25^+^ regulatory T cells to release soluble fibrinogen-like protein 2 (sFGL2)	AAD10825.1	Inhibit macrophages from releasing monocyte chemoattractant protein-1 (MCP-1) for substantial recruitment of natural killer/natural killer T cells, production of interferon-γ and activation of the c-Jun N-terminal kinase phosphorylation in the toll-like receptor two signaling pathway	Intra-erythrocytic stages	In vitro and in vivo	(Fu et al. 2020)
*Plasmodium falciparum*	Upregulation of some variant surface antigen (VAR2CA)	PF3D7_1200600	The VSA promotes protection from macrophage phagocytosis (CD36) and interaction with the immune inhibitory receptor (LILRB1)	Intra-erythrocytic stages	In vivo	(Chew et al. 2022)
*Plasmodium berghei*	*Plasmodium berghei-***release factor (*Pb*TIP)—**T-cell immunomodulatory protein (TIP), expressed on the surface	PbANKA_124360.0	Upregulation expression of *Pb*TIP caused a reduction in the production of inflammatory cytokines and promoted the expression of anti-inflammatory cytokines such as TGF-β and IL-10	Intra-erythrocytic stages	In vitro	(Kalia et al. 2021)

It was recently shown that MPs, on entry into the erythrocyte of the host, use a protein known as the erythrocyte binding protein 175 (EBA-175), having a band at 175 kDa on the SDS-PAGE, to bind to the glycophorin A (a glycoprotein found on human erythrocytes) (Jaskiewicz et al. 2019). However, a recent study has reported that EBA-175 dissociates from the merozoites once they enter the erythrocyte, a phenomenon known as “antigen shedding.” Furthermore, the shed EBA-175 facilitates the clustering of erythrocytes to form rosettes (Paing et al. 2018). Consequentially, iRBCs within the clusters are masked from attack by the immune cells and pathologically lead to constriction of the blood vessels, resulting in several malaria complications (Okagu et al. 2022). Another study reported that *P. berghei* NK65 (as well as other *Plasmodium* species) secrete extracellular vesicles (EVs) to foster their survival and infectivity (Demarta-Gatsi et al. 2019). More so, two proteins — histamine-releasing factor (HRF) and the elongation factor 1α (EF-1α) — were reported to be associated with EVs of *P. berghei* NK-65 and were implicated with immunosuppression of CD4^+^ T cells during the blood stages of the parasite infection (Demarta-Gatsi et al. 2019). In an in vivo experiment, the EV proteins inhibited the ovalbumin-specific delayed-type hypersensitivity response. In contrast, in a cell line experiment, the proteins were discovered to dephosphorylate and inactivate essential molecules (such as PLCγ1, Akt, and ERK) on the pathway of the T-cell receptor signaling cascade (Demarta-Gatsi et al. 2019). In conclusion, long-lasting immune protection and memory were achieved by immunizing Swiss Webster mice with HRF and EF-1α (Demarta-Gatsi et al. 2019).

*P. falciparum* was recently discovered with the ability to acquire the host plasma zymogen — plasminogen which is activated into a serine protease, plasmin by urokinase-type and tissue-type activators (Reiss et al. 2021). Plasmin showed a high affinity to degrade fibrinogen, and C3 and C5 complements are known to mount an immune response against the parasite. Moreover, a reasonable concentration of plasminogen was discovered to be concentrated as the probable entry site of merozoites (Reiss et al. 2021). Another study reported that during the blood stage of malaria, the parasite induces CD4^+^Foxp3^+^CD25^+^ regulatory T cells to release a fibrinogen-like protein 2 (sFGL2), resulting in immunosuppression, thereby enhancing the infection (Fu et al. 2020). This sFGL2 was discovered to inhibit the activities of macrophages by preventing the release of MCP-1, which is responsible for the signaling and recruitment of natural killer/natural killer T cells and INF-γ (Fu et al. 2020). Specifically, sFGL2 altered and inhibited c-Jun N-terminal kinase phosphorylation in the toll-like receptor 2 signaling pathway of macrophages and prevented the release of MCP-1 from the FcγRIIB receptor (Fu et al. 2020).

It was also recently discovered that microRNA (miRNA) plays a crucial role in fostering immunological evasion by malaria parasites (Acuña et al. 2020). A study by Dieng et al. (2020) reported from an integrative genomic analysis that *P. falciparum* expresses high polymorphic microRNA (about 1376 genetic variants expressing 34 miRNA), among which miR-16-5p, miR-15a-5p, and miR-181c-5p foster lymphocyte apoptosis. This process, therefore, promotes a survival advantage for the parasites.

Other novel evading mechanisms of a *Plasmodium* parasite in the invertebrate mosquito host have been recently reported. Glutaminyl cyclase, initially known for its post-translational modification of the N-glutamine or glutamic acid into pyroglutamic acids, was recently reported to play an exciting role in preventing the recognition and melanization of the parasite by the immune systems and hemocoel of the mosquito, respectively (Kolli et al. 2021). Hence, it is valuable for the parasite’s surface (sporozoites) to be post-translationally modified with glutaminyl cyclase to effectively replicate and survive in the invertebrate parasites (Kolli et al. 2021). Other proteins such as PIMMS43 (*Plasmodium* infection of the mosquito midgut screen 43) have been reported to be present on the surface of parasites (predominantly from Africa) and responsible for fostering the transmission of the parasite from mosquito to human after a blood meal (mosquito bites). Inhibition of PIMMS43 by complete gene knockdown and blocking by antibodies inhibited malaria parasites (Ukegbu et al. 2020). In conclusion, understanding the proper function of some of these proteins and genes as regards immune evasions by the *Plasmodium* parasite could foster the discovery of a more potent antimalarial drug or vaccine against the infection (Wilson et al. 2019).

## Conclusions

This review provided an exciting discussion of current knowledge on how the immune system mounts an attack on MPs upon invasion of the skin, live, and erythrocytes, as well as the cellular and molecular mechanisms via which MPs escape from the wrath of the host immune system. Despite the array of publications on malaria biology, curbing the burden of the disease has remained a big challenge, warranting more research efforts towards improving our understanding of other pathways yet to be unraveled through which parasites evade the host immune system. A competent and effective malaria vaccine remains the major hope for eradicating malaria or at least reducing the burden to the barest minimum. However, discovering a clinically effective malaria vaccine is highly dependent on a comprehensive understanding of malaria biology. However, boosting individuals’ immune systems in malaria-endemic regions, as suggested previously (Okagu et al. 2022), will make them immunocompetent to produce memory cells upon primary infection to fight malaria parasites during secondary infection. It is well-known that MPs, through the expression of *PfEMP1*, bind to CD36 and increase the expression of pro-angiogenic and endothelial activation molecules such as vascular endothelial growth factor (VEGF)–A and its receptor vascular endothelial growth factor receptor 2 (VEGFR2), c-reactive protein (CRP), platelet factor–4, intercellular adhesion molecule (ICAM)–1, and von Willebrand factor (vWF), endothelial protein C receptor (EPCR), leading to their sequestration into tissues to cause tissue damage while escaping from splenic clearance (Furuta et al. 2010; Park et al. 2012; Turner et al. 2013; Canavese and Spaccapelo 2014; Tuikue Ndam et al. 2017; Björkman 2018; Dos-Santos et al. 2020; Frimpong et al. 2021). Future studies should consider these the potentials of using these molecules as both diagnostic marker of asymptomatic malaria and therapeutic targets. Future research should consider developing small biocompatible molecules that can prevent the interaction of MP-originating rosetting ligands with the membrane of erythrocytes, to inhibit their escape from host immune cells. Another strategy is to develop cocktail vaccines that can bind to different malaria parasite proteins needed for invasion into host cells allowing phagocytes to destroy them. Other possible strategies include multitargeted small molecule adjuvants that hamper various channels through which malaria parasites escape host immune attack while equipping the host soldiers, especially the early immune responders such as NK cells and Kupffer cells, at the early stage of infection.

## Data Availability

All data reported in this work are available.
